# Basic Properties of MgAl-Mixed Oxides in CO_2_ Adsorption at High Temperature

**DOI:** 10.3390/ma16165698

**Published:** 2023-08-19

**Authors:** Dylan Chaillot, Vincent Folliard, Jocelyne Miehé-Brendlé, Aline Auroux, Liva Dzene, Simona Bennici

**Affiliations:** 1 Institut de Sciences des Matériaux de Mulhouse, CNRS, Université de Haute-Alsace, Université de Strasbourg, 15 Rue Jean Starcky, 68057 Mulhouse CEDEX, France; dylan.chaillot@uha.fr (D.C.); jocelyne.brendle@uha.fr (J.M.-B.); liva.dzene@uha.fr (L.D.); 2 Institut de Recherches sur la Catalyse et l’Environnement de Lyon, Université Lyon 1, CNRS, UMR 5256, IRCELYON, 2 Avenue Albert Einstein, F-69626 Villeurbanne, France; vincent.folliard@ircelyon.univ-lyon1.fr (V.F.); aline.auroux@ircelyon.univ-lyon1.fr (A.A.)

**Keywords:** Layered Double Hydroxides, mixed oxides, basicity, CO_2_ adsorption, adsorption calorimetry

## Abstract

The increase of consciousness towards global warming and the need to reduce greenhouse gas emissions lead to the necessity of finding alternative applications based on easy-to-use materials in order to control and reduce global CO_2_ emissions. Layered Double Hydroxides (LDHs) and LDH-derived materials are potentially good adsorbents for CO_2_, thanks to their low cost, easy synthesis, high sorption capacity, and surface basicity. They have been intensively studied in CO_2_ capture at high temperature, presenting variable sorption capacities for MgAl LDHs with the same composition, but prepared under different synthesis conditions. The ambient temperature coprecipitation synthesis method is an attractive one-step procedure to synthesize LDHs under mild conditions, with low energy consumption and short synthesis time. The present study is based on the synthesis and characterization of hydrotalcites by a mild-conditions coprecipitation process and the production of derived mixed oxides to be used as CO_2_ adsorbents. A critical comparison to similar materials is reported. Moreover, the effect of the surface basicity of the derived mixed oxides (measured by adsorption calorimetry) and the CO_2_ sorption capacity are discussed, showing a linear correlation between the amount of weak and very strong basic sites and the CO_2_ adsorption behavior.

## 1. Introduction

Global warming, pollution, and health concerns often derive from the environmental impacts caused from industrial activities. The need to decrease the actual massive use of fossil fuels boosts the research on alternative solutions for limiting the emissions of CO_2_. CO_2_ is among the main greenhouse gases, and its contribution has been estimated to more than 60% of the total global warming. The Kyoto Protocol and international conventions intend to reduce global emissions by 50% with respect to those measured in 2006 by 2050. Consequently, the necessity of finding easy-to-use materials for CO_2_ adsorption becomes more than urgent. 

Several methods for the safe control and disposal of CO_2_ emissions have been widely studied [1]. For example, steam reforming of hydrocarbons is the most suitable process for hydrogen production, but it releases high amounts of carbon dioxide [2,3,4] that must be adsorbed. An appropriate CO_2_ adsorbent should satisfy the following criteria: a low cost, a fast kinetic, a high adsorption capacity and selectivity, and a high thermal and chemical stability for several adsorption cycles [1,5,6,7]. Clay minerals are potentially good adsorbents, but they are generally stable only up to 200 °C [8,9,10]. Due to the loss of interlayer water (dehydration), the irreversible degradation of the structure takes part at higher temperatures than 200 °C. Among all the materials tested for CO_2_ capture and storage, zeolites [11,12] and LDHs [7,13,14,15] have been widely studied thanks to their high surface area, developed pore structure, and high charge density [5]. Zeolites present high charge density and tunable pore size [16,17], while LDH-based materials represent an interesting alternative material thanks to their easy synthesis and the tunability of their chemical composition. Various preparation routes of LDH-based materials have been considered for synthesizing materials for CO_2_ capture. In this frame, the impregnation of commercial hydrotalcites [18], the synthesis of tunable Mg/Al LDHs [14,15,19], and the formation of mixed oxides derived from their calcination [6,20,21,22] have been reported.

Layered Double Hydroxides are lamellar materials made of stacked octahedral sheets containing a mixture of divalent and trivalent cations, according to the following structural formula:

[M^2+^_1−x_M^3+^_x_(OH)_2_]^x+^(A^n−^)_x/n_.yH_2_O (1)

where M^2+^ is a divalent metal cation (Mg^2+^, Ca^2+^, or Zn^2+^, for example), M^3+^ is a trivalent metal cation (Al^3+^, Fe^3+^, or Co^3+^, for example), and A^n−^ is a compensating anion (such as Cl^−^, CO_3_^2−^, or NO_3_^−^).

LDHs are generally compared to conventional clay-like crystalline structures and are named sometimes “anionic clay” due to the positive charge deficit that is compensated by anions in the interlayer space. Hydrotalcite is a particular type of LDH made of magnesium and aluminum cations with a fixed Mg/Al molar ratio equal to 3.

Regarding their widest application, CO_2_ adsorption is strongly related to the number and the strength of surface basic sites [6,23,24]. Even if they present a lower CO_2_ adsorption capacity, when compared to other more conventional sorbents [25,26,27,28], the high presence of water molecules can increase their CO_2_ adsorption capacity [29]. However, these materials are significantly more active for CO_2_ adsorption after thermal decomposition, to form basic mixed oxides [30,31]. Thanks to the presence of Al^3+^ cations in the lattice, hydrotalcite-derived materials exhibit good performances for the adsorption of CO_2_ [32,33]. The parameters that play a role in improving the surface acidity and basicity in LDH-derived materials are the presence of compensating anions in the interlayer space [34,35,36], the synthesis method [37,38], and the temperature of the treatment [34]. The tunability of the surface acid/basic properties is an important parameter, depending on the target application. As an example, the removal of Cl^−^ by anion exchange leads to an increase in the surface basicity, as reported by Tichit et al. [31]. The authors concluded that the Cl^−^ anions block the basic sites of hydrotalcites. The surface basicity of these materials has been improved by adding alkali salts and by increasing the Mg/Al molar ratio [39]. In addition, the presence of transition metals in the structure of the hydrotalcites tends to increase the surface acidity of the corresponding hydrotalcite-derived mixed oxides, as reported by Pavel et al. [40]. Moreover, the possibility to be regenerated [41,42] and the adsorption reversibility [43] of the hydrotalcite materials are also important points to enhance the efficiency of these sorbents in industrial adsorption units.

Coprecipitation in mild conditions is a more recent synthesis route of choice to prepare hydrotalcites in a shorter time than that employed in the conventional coprecipitation method [35,44,45]. This coprecipitation method consists of dissolving the inorganic salts containing the divalent and trivalent metal cations in a solvent, generally ethanol or water, and increasing the solution pH by adding a basic solution. The base addition promotes the condensation reaction. This procedure allows for obtaining compounds without secondary phases in a shorter time and with lower energy consumption than the conventional coprecipitation method, but with lower crystallinity [46,47,48].

In the present study, a series of hydrotalcite precursors (LDHs), prepared by the mild-conditions coprecipitation method, was firstly characterized by X-ray Diffraction, solid-state ^27^Al Nuclear Magnetic Resonance spectroscopy, X-ray fluorescence spectroscopy, BET surface area measurements, and Thermogravimetric Analyses. Due to the difficulty of having insights on the condensation mechanism, it is still not clear why differences in the acid/base properties (even if very small) can be measured. Further investigation is then strongly needed on this point. The present manuscript aims to give some interesting insight to contribute to this debate. At different preparation pH values, the condensation reaction to form the hydrotalcites might take part following different pathways (not yet elucidated up to now) to form a final product with slightly different ratios among the Mg- and Al-containing phases. Segregation of Mg and Al hydroxide can also take over, and, even if not detectable by the techniques employed, drive the acid-basic properties of the final materials. In the present paper, differently to that reported in the most part of the already published articles, the surface basicity (the key property for CO_2_ adsorption) of the calcined materials (mixed oxides) was probed by SO_2_ adsorption calorimetry. In a second time, the CO_2_ sorption capacities of the mixed oxides have been measured in order to compare their performances with similar materials reported in the literature and find correlations between the physico-chemical characteristic and the surface basicity. CO_2_ adsorption was here carried on at the temperature of 200 °C to verify the compatibility of the prepared material with industrial processes where a high adsorption temperature is required (among others in the so-called “carbon capture and utilization process”, where CO_2_, once captured, is utilized as a feedstock and converted catalytically into methane or methanol).

## 2. Materials and Methods

### 2.1. Synthesis of the Layered Double Hydroxides

The synthesis of the MgAl LDHs precursors involves magnesium chloride and aluminum chloride as sources of the atoms constituting the material framework. Magnesium chloride hexahydrate MgCl_2_.6H_2_O (Sigma-Aldrich, BioXtra, Burlington, MA, USA, ≥99.0 wt%), aluminum acetylacetonate Al(C_5_H_7_O_2_)_3_ (Sigma-Aldrich, ReagentPlus^®^, 99 wt%), aluminum chloride hexahydrate AlCl_3_ · 6H_2_O (Fluka Analytical, ≥99.0 wt%), ethanol CH_3_CH_2_OH (Carlo Erba Reagents, Emmendingen, Germany, ≥97 wt%), sodium hydroxide NaOH (Carlo Erba Reagents, 99.9 wt%), and deionized water (18.2 MΩ·cm) were used.

At first, the required amounts of magnesium and aluminum precursors (to fix the Mg/Al molar ratio at 3 according to the general structural formula of the hydrotalcites: [Mg_6_Al_2_(OH)_16_] (A^n−^)2.yH_2_O), where A^n−^ are the Cl^−^ and C_5_H_7_O_2_^−^ that were dissolved into 50 mL of ethanol under stirring. The Mg/Al molar ratios were calculated according to the amounts of each reactant and their molar masses. Then, an aqueous solution of sodium hydroxide (NaOH) 1M was added to adjust the pH at 11 for the HTCL-1 and HTCL-3 samples and 10 for the HTCL-5 sample (see Table 1). The mixture was left under stirring at room temperature for 24 h. The solid was recovered by centrifugation at 11,000 rpm for 10 min, washed three times with 30 mL of ethanol, and finally dried for 24 h in an oven at 60 °C. The samples were then labeled “HTLC-x”, where x is the number of the sample. Two different precursors (AlCl_3_.6H_2_O and Al(C_5_H_7_O_2_)_3_) were used in order to verify if the substitution of Cl^−^ with C_5_H_7_O_2_^−^ would lead to an increase of the surface basicity; indeed, in Ref. [31], the authors concluded that the Cl^−^ anions block the basic sites of hydrotalcites. Moreover, two samples obtained using AlCl_3_.6H_2_O as the Al source were prepared by adding different amounts of NaOH solution (28 mL for HTLC-1 and 16 mL for HTLC-5) in order to verify if the different conditions of condensation (at pH 11 and 10, respectively) will lead to different CO_2_ adsorption properties of the derived oxides.

Finally, the powders of hydrotalcite precursors were calcined at 450 °C for 5 h in an oven. The calcined samples (hydrotalcite-derived mixed oxides) were labeled “HTLC-x CAL”, x corresponding to the number of the sample. The resultant oxides were used for CO_2_ adsorption in this study.

### 2.2. Physico-Chemical Characterization Techniques

The structural properties of the synthesized samples were investigated by X-ray Diffraction using a Panalytical X’Pert PRO MPD diffractometer with Cu Kα radiation (λ = 1.5418 Å), and performed from 2 to 70° 2θ, with a step of 0.017° 2θ and a time per step of 218 s, with a total time for acquisition of 1 h 15, on randomly oriented powder samples. Each reflection is associated with a distance between planes according to the Bragg’s Law: 2d_hkl_.sin(θ) = n.λ (where d_hkl_ corresponds to the distance of the plane (hkl), θ the diffraction angle, λ the Kα radiation used (1.5418 Å), and n the periodicity index).

Solid-state ^27^Al MAS NMR spectroscopy spectra were obtained using a Bruker AVANCE II 400 MHz spectrometer at 104.3 MHz with magic angle spinning (MAS). The samples were packed in a 2.5 mm diameter cylindrical rotor, spun at a spinning frequency of 25 kHz, and recorded for 8 h. A short delay time of 1 s and a 4 µs single pulse were used.

Thermogravimetric analysis (TGA) of the synthesized samples was performed using a Mettler-Toledo TGA/DSC1 LF1100 apparatus, in alumina sample holders, under argon, with a flow rate of about 100 mL·min^−1^ from 25 to 600 °C and a heating rate of 5 °C·min^−1^. An empty sample holder was recorded as reference to correct the baseline deviation.

N_2_ adsorption–desorption isotherms were obtained on a Micromeritics ASAP 2420 apparatus at −196.15 °C. The samples were degassed at 100 °C for 15 h before the measurements. The data were analyzed by means of the software MicroActive 5.02.

### 2.3. Basicity Measurement by SO_2_ Adsorption Calorimetry

The surface basicity of the samples was determined by adsorption microcalorimetry of SO_2_ at 150 °C. The experimental set-up was constituted of a Setaram C80 heat-flow calorimeter linked to a homemade volumetric apparatus equipped with a Barocel Capacitance manometer, for pressure measurements. Approximately 80 mg of the samples was preheated in a quartz cell by heating overnight under vacuum at 350 °C. The differential heat of adsorption was measured as a function of the coverage by repeatedly sending small doses of the gas probe onto the sample until an equilibrium pressure of 0.5 Torr was reached. The sample was then outgassed for 40 min at the same temperature, and a second adsorption run was performed at 150 °C on each sample, until an equilibrium pressure of approximately 0.2 Torr. The difference between the amount of the probe gas adsorbed during the first and the second runs represents the irreversibly adsorbed amount (V_irr_) of the probe gas, which provided an estimation of the number of strong basic sites.

### 2.4. CO_2_ Adsorption Tests

A Setaram Sensys thermogravimetry–differential scanning calorimetry (TG-DSC) apparatus has been used to measure the adsorption of CO_2_. Samples were pretreated in situ at 300 °C under pure N_2_ flowing at 20 mL·min^−1^. Then, the temperature was decreased and stabilized to the adsorption temperature (200 °C) always under N_2_ flow. Once the mass stabilized, the gas was switched to CO_2_ with a constant flow of 20 mL·min^−1^ and maintained during the CO_2_ sorption experiments for 4 h until complete saturation of the samples. The mass losses and uptakes were measured in order to respectively evaluate the materials CO_2_ adsorption capacity.

## 3. Results and Discussion

### 3.1. Characterization of the Hydrotalcite Precursors

**Figure 1:**
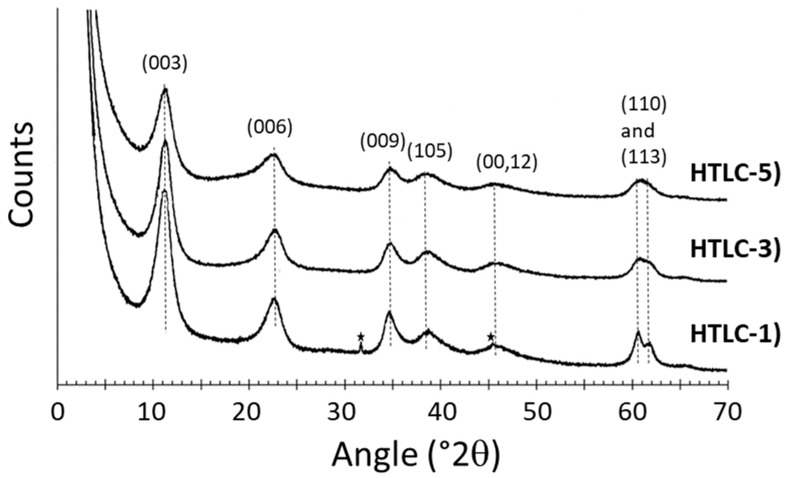
X-ray Diffractograms of the hydrotalcite samples. The star symbols correspond to NaCl impurities.

Due to their 2-dimensional structure, the X-ray diffraction patterns of LDHs generally show (00ℓ) reflections that allow an estimation of the basal spacing (d_003_) and the c-cell parameter c = 3 × d_003_, corresponding to the interlayer distance plus the thickness of a single layer three times in case of rhombohedral symmetry [49]. Thus, (003) and (110) reflections are respectively related to the size of the unit-cell, c = 3 × d_003_, and the metal–metal interatomic distance in a sheet, a = 2 × d_110_. Figure 1 displays the X-ray diffraction patterns of the precursors synthesized in this study. Seven main reflections are observed, 11.2, 22.7, 34.6, 38.7, 45.5, 60.6, and 61.8 °2θ, which correspond to distances of 7.9 (d_003_), 3.9 (d_006_), 2.6 (d_009_), 2.3 (d_105_), 2 (d_00,12_), 1.52 (d_110_), and 1.49 (d_113_) Å, respectively, that, according to the Bragg’s law, were detected on the sample HTLC-1 that was the only sample presenting NaCl impurities (not completely removed during the post-preparation washing procedure). These positions correspond to MgAl LDHs, also called hydrotalcites, despite the broadness of the peaks related to a lower structural organization (crystallinity) due to the coprecipitation synthesis process (short synthesis time without heating). This phenomenon is observed in the samples HTLC-3 and HTLC-5 by the overlapping of the d_110_ and d_113_ planes, which is related to the reactants used.

**Figure 2:**
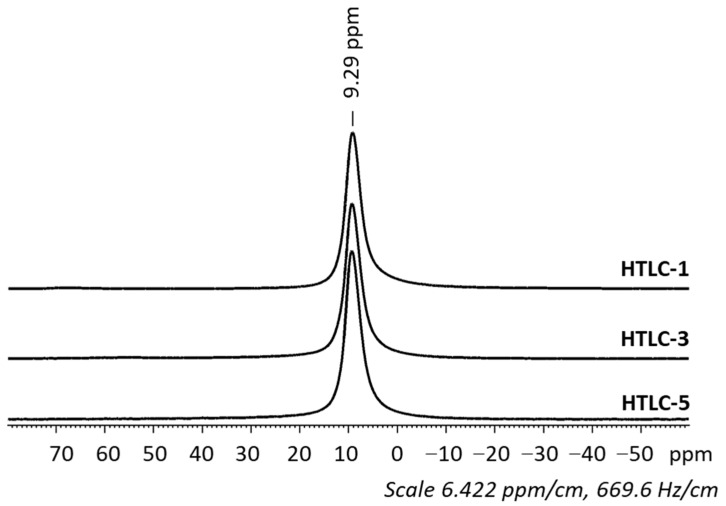
Solid-state ^27^Al Nuclear Magnetic Resonance spectra of the non-calcined samples.

Then, solid-state ^27^Al Nuclear Magnetic Resonance (NMR) spectroscopy was performed to probe the local environment and the coordination state of ^27^Al into the synthesized LDHs. According to Figure 2, a single resonance can be observed around 9.3 ppm that corresponds to octahedral aluminum (Al VI coordination state). The configuration of aluminum in an octahedral environment is thus confirmed by the presence of this resonance, observed in all the synthesized samples.

**Figure 3:**
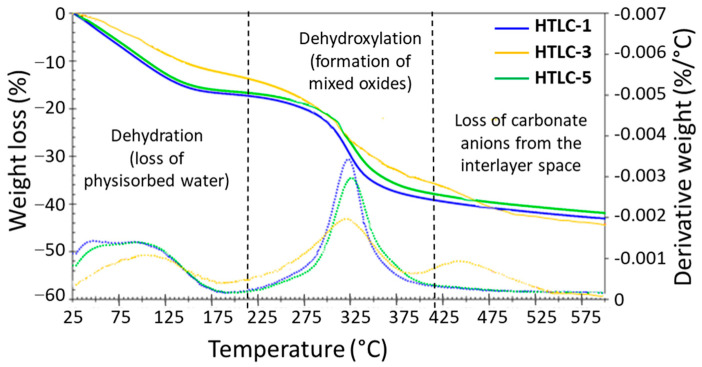
Thermogravimetric analyses of the hydrotalcite samples, recorded from 25 to 600 °C. The weight losses are shown in solid lines and their derivatives in dotted lines.

Then, Thermogravimetric Analyses (TGA) have been performed by increasing the temperature under argon flow from 25 to 600 °C, as shown in Figure 3, in order to evaluate the thermal stability of the precursors and the evolution of the hydrotalcite into the final mixed oxides. All samples present the typical thermogravimetric (left axes) and derivative thermogravimetric profile (right axis) of hydrotalcites decomposition. The samples exhibit similar profiles with a first weight loss of about 13 to 16.4 wt% between 25 and 200 °C, and related to the dehydration of the LDHs (loss of the surface water). A second weight loss of about 25.5 to 31.3 wt% between 200 and 600 °C was assigned to the dehydroxylation of the lamellar materials to form the corresponding mixed oxides. The third weight loss, observed in the sample HTLC-3 between 390 and 550 °C, was related to the departure of CO_2_ (derived from C_5_H_7_O_2_^−^ anions) present in the interlayer space. Compared to similar materials in the literature [50,51,52,53], Table 2 shows that the MgAl LDHs sample here prepared are as thermally stable as conventional LDHs that are mainly synthesized by methods with longer synthesis durations.

### 3.2. Characterization of the Hydrotalcite-Derived Mixed Oxides

The mixed oxides obtained after calcination of the hydrotalcite precursor at 450 °C for 5 h under air were also characterized.

**Figure 4:**
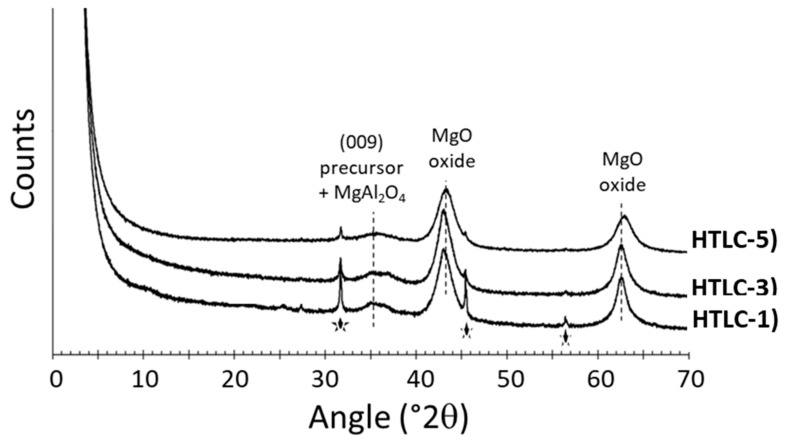
X-ray Diffractograms of the hydrotalcite-derived mixed oxides. The star symbols correspond to NaCl impurities.

The structural modifications due to the calcination process were evidenced by XRD, reported in Figure 4. Despite the presence of NaCl impurities observed in the precursors, the layered structure of the hydrotalcite precursor clearly collapsed after calcination. An amorphous phase characterized by broad peaks was observed. The peaks centered at 35.1, 37.2, 43.4, and 63° 2θ corresponded respectively to the precursor (35.1° 2θ), MgAl_2_O_4_ oxide (37.2° 2θ), and MgO oxide (43.4 and 63° 2θ). These structural changes are associated to the dehydroxylation of the materials observed by TGA (between 200 and 600 °C) and the formation of mixed oxides.

**Figure 5:**
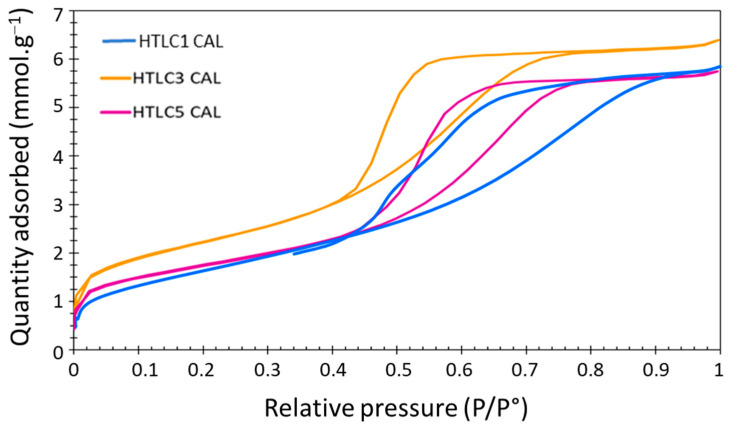
N_2_-adsorption isotherms for the three investigated samples.

Then, the nitrogen adsorption/desorption isotherms were collected to determine the specific surface area and the microstructure of the calcined hydrotalcite samples (see Figure 5). Despite the different Al-sources used for the synthesis of the hydrotalcite precursors (respectively, acetylacetonate and chloride for HTLC-3 and HTLC-1), all calcined samples presented isotherms of type IVa and hysteresis loops of type H2b, characteristic of mesoporous adsorbents, according to the IUPAC classification [54]. Such a type of hysteresis is characteristic of a complex and interconnected pore structure. The specific surface areas have been calculated by the BET method. The samples showed high surface areas, respectively, of 138, 180, and 139 m²·g^−1^ for the HTCL-1 CAL, HTCL-3 CAL, and HTCL-5 CAL, with medium pore volumes in the 0.20–0.22 mmol·g^−1^ range in all the calcined samples. These results are in agreement with the ones reported in the literature for similar mixed oxides [55,56,57]. The samples prepared with the precursor containing the acetylacetonate anion lead to a material with a slightly higher surface area. On the other hand, the amount of OH^−^ added had little effect on the surface area of the calcined samples, despite the differences observed for non-calcined samples in terms of crystallinity.

#### 3.2.1. SO_2_ Adsorption Calorimetry

Adsorption calorimetry is the most adapted technique for determining the concentration (from the adsorption volumetric isotherms), strength, and strength distribution (using the differential heat of adsorption as a function of coverage) of the basic sites [58] involved in CO_2_ adsorption. Then, the hydrotalcite-derived mixed oxides surface basicity has been probed by adsorption calorimetry of SO_2_.

**Figure 6:**
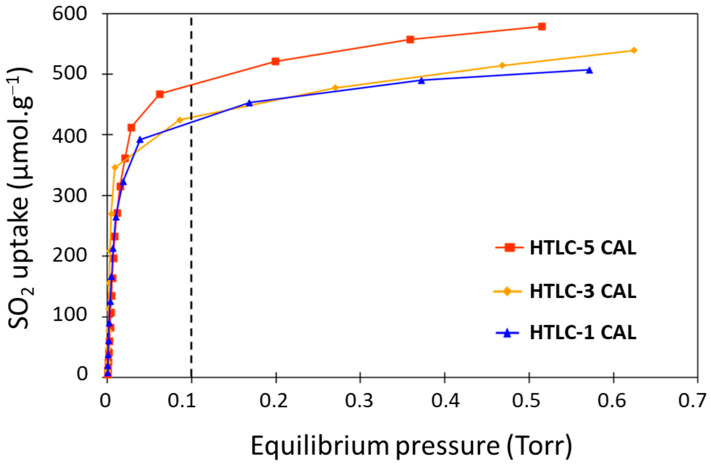
SO_2_ adsorption isotherms of the calcined hydrotalcite samples.

Figure 6 exhibits the SO_2_ adsorption isotherms of the calcined hydrotalcite samples HTLC-1 CAL, HTLC-3 CAL, and HTLC-5 CAL. Some differences can be observed between the samples: HTLC-5 CAL has a higher SO_2_ uptake (up to 578 µmol·g^−1^ at 0.5 Torr), which is associated to a higher basicity than the other samples. After a fast uptake at low equilibrium pressure (p < 0.1 Torr) due to the adsorption on the strongest sites, the SO_2_ uptake reaches a plateau around 550 µmol·g^−1^, even increasing the equilibrium pressure. Despite the difference in terms of specific surface area of the samples HTLC-1 CAL and HTLC-3 CAL (138.3 and 174.8 m².g^−1^, respectively), their SO_2_ adsorption isotherms are quite similar.

**Figure 7:**
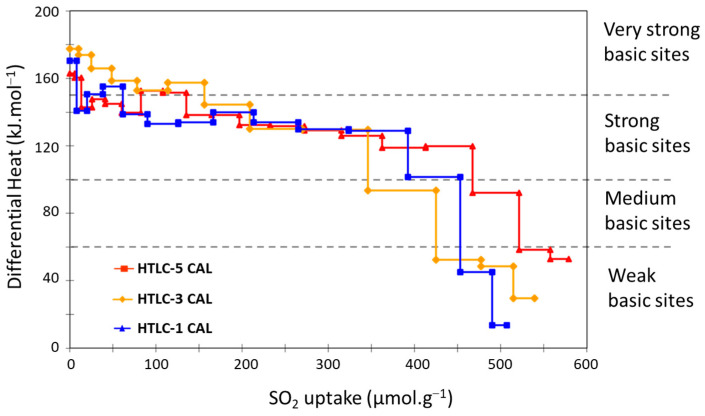
Differential heats of SO_2_ adsorption of the calcined hydrotalcite samples.

In Figure 7, the SO_2_ differential heat curve shows a pseudo plateau placed at around 140 kJ·mol^−1^, indicating the homogeneity in the strength of the basic sites present in the mixed oxide. This is not a common result for such kinds of samples, which generally present a more heterogeneous strength site distribution [59,60]. For more insights: at low coverage, the curves present a few points characterized by a relatively high adsorption heat (between 150 and 180 kJ·mol^−1^, which can be connected to the presence of Lewis adsorption sites (very strong sites)). At a coverage higher than 50 µmol·g^−1^, a plateau (that ends up, respectively, around 350, 400, and 450 µmol·g^−1^ for HTLC-3 CAL, HTLC-1, and HTLC-5) can be ascribed to SO_2_ adsorption on relatively strong sites and characterized by an almost constant heat. The sites represented by this plateau correspond to homogeneous BrØnsted sites (most probably –OH groups). After the plateau, a region characterized by steep decreasing in the differential heat curve can be observed and assigned to the presence of a small number of heterogeneous sites (probably of Lewis type) [60]. The last part of the curve corresponds to the reversible adsorption domain (physisorption of the SO_2_ probe) or to very weak Lewis acid sites.

**Figure 8:**
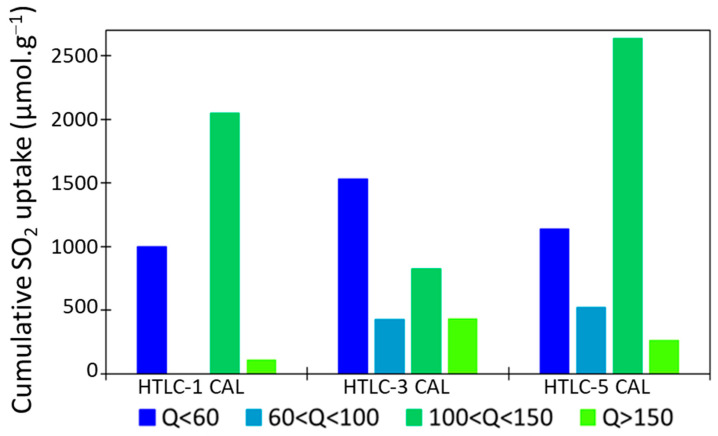
Probe uptake of the calcined samples depending on the range of energy.

More in detail, the basic sites can be divided into four groups according to their strength: very strong (above 150 kJ·mol^−1^), strong (in the range 100–150 kJ·mol^−1^), medium (in the range 60–100kJ·mol^−1^), and weak (below 60 kJ·mol^−1^). The distribution is reported in Figure 8.

Some differences can be observed among the samples. The sample HTLC-5 CAL, for example, presents the highest SO_2_ adsorption and the highest ratio of strong basic sites. As a general observation, the three samples present a low proportion of very strong sites (Q > 150 kJ·mol^−1^). This is a positive feature in view of the application of these materials as CO_2_ adsorbents, because the strong site can lead to the formation of surface carbonates that react in an irreversible way with CO_2_.

The amount of SO_2_ adsorbed is not directly related to the specific surface area of the materials: the sample HTLC-3 CAL that exhibits the highest surface area (179.9 m²·g^−1^) presents an intermediate total SO_2_ adsorption value of 539.2 µmol·g^−1^ at 0.5 torr. The highest SO_2_ sorption value is observed in the sample HTLC-5 CAL that exhibits a lower surface area (139.4 m²·g^−1^).

#### 3.2.2. CO_2_ Adsorption Tests

**Figure 9:**
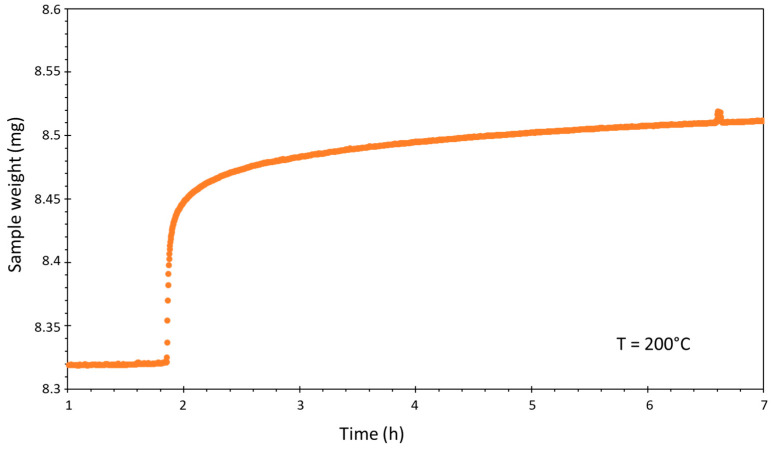
CO_2_ uptake curve for the sample HTLC-3 CAL, reported as an example.

**Figure 10:**
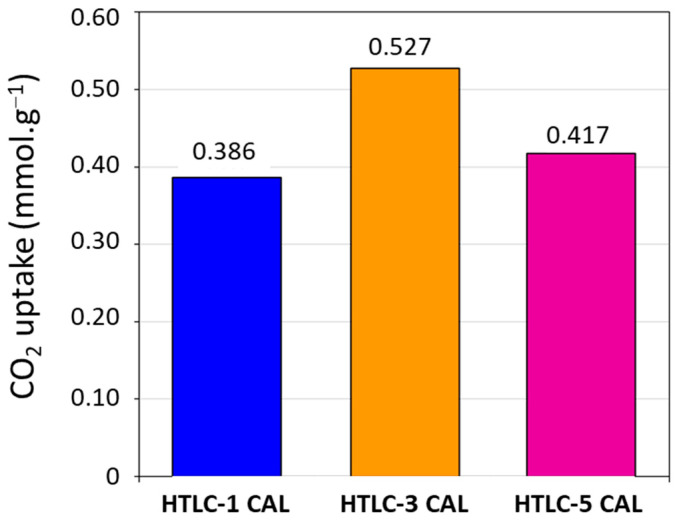
Quantity of CO_2_ adsorbed by the calcined hydrotalcite samples (HTCL-1 CAL in blue, HTCL-3 CAL in orange, and HTCL-5 in fuchsia).

Finally, a screening of the adsorption capacity of CO_2_ has been performed on the calcined samples. As an example, the CO_2_ uptake curve (performed at 200 °C) and the pretreatment and adsorption temperature program are shown in Figure 9 for the sample HTLC-3 CAL. The results are shown in Figure 10, which shows that the studied samples exhibit CO_2_ adsorption capacities between 0.38 and 0.53 mmol·g^−1^. The reproducibility of the experiments was verified and the error on the measurements estimated at ±0.002 mmol∙g^−1^.

Table 3 compares these adsorption values with similar materials found in the literature, and proves that the samples here synthesized in mild conditions are as efficient (under similar analyses conditions) as other hydrotalcites synthesized by other methods for the adsorption of CO_2_. They present adsorption capacities around 0.5 mmol·g^−1^, without the need of additional treatments or structural modifications.

Sample HTLC-3 CAL, which exhibits a large amount of weak basic sites (mainly below 60 kJ·mol^−1^) and the highest specific surface area (179.9 m²·g^−1^), shows the highest CO_2_ adsorption capacity (0.527 mmol·g^−1^).

**Figure 11:**
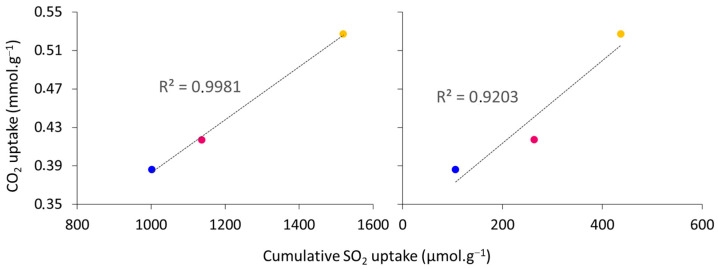
Linear correlations between the concentrations of weak (below 60 kJ·mol^−1^) and very strong (above 150 kJ·mol^−1^) energy sites and the CO_2_ adsorption capacities of the calcined samples (HTCL-1 CAL in blue, HTCL-3 CAL in orange, and HTCL-5 in fuchsia).

The interaction of CO_2_ with the basic sites of the material depends on their strength. Typically, low-energy basic sites (Q < 60 kJ·mol^−1^), measured by SO_2_ adsorption calorimetry, correspond to weak physisorption sites and are related to reversible sorption. On the other hand, high-energy basic sites (Q > 150 kJ·mol^−1^) are related to strong chemisorption. Thus, low-energy sites remain accessible after thermal regeneration, which is important for the reusability of the materials. High-energy sites cannot be regenerated and are irreversibly poisoned by acid/amphoteric probes, such as CO_2_. In the present sample series, a very good linear correlation between the concentration of low- (Q < 60 kJ·mol^−1^) and very high-(Q > 150 kJ·mol^−1^) energy sites and the CO_2_ adsorption capacity can be noticed. This observation is illustrated in Figure 11: the higher the concentration of these sites, the higher the sorption capacity of the material. This demonstrates that the sorption capacity of these hydrotalcites is strongly influenced by the type of sites. Even if the correlation with the amount of weak and very strong adsorption sites is linear, very strong sites can irreversibly adsorb CO_2_. Then, the reusability of the sorbents might be maximized when the sample presents a high number of weak adsorption sites and a limited amount of strong energy sites. This point will need further investigation prior to the application of such materials in real applications.

## 4. Conclusions

Layered Double Hydroxides, especially hydrotalcites, are very interesting adsorbents for carbon dioxide thanks to their lamellar structure and strong basicity. In this work, hydrotalcites with a Mg/Al molar ratio of 3 have been successfully synthesized by coprecipitation in mild conditions and characterized by complementary physico-chemical techniques. The influence of the pH and of the Al source has been investigated. Their thermal stability has been evaluated by DTA/TGA, showing a major structural change around 325 °C, due to the formation of mixed oxides. These oxides show a high specific surface area (up to 180 m²·g^−1^ for the sample prepared starting from MgCl_2_._6_H_2_0 and Al(C_5_H_7_O_2_)_3_), as well as a surface basicity (high quantity of medium and weak basic sites) adapted to the reversible adsorption of CO_2_. The calcined hydrotalcites exhibit CO_2_ adsorption capacities of around 0.5 mmol·g^−1^, a value similar to those reported in the literature for samples generally synthesized by conventional coprecipitation methods. The use of the Al(C_5_H_7_O_2_)_3_ gave rise to the sample with the highest specific surface area and containing the optimum quantity of the basic site of adapted energy (measured by adsorption microcalorimetry) for CO_2_ adsorption.

On the other hand, the modification of the pH of condensation during synthesis did not impact the main microstructural properties of the samples that presented the same specific surface area and pore volume for the synthesis performed at pH = 10 and 11. Only the surface basicity was modified; the calcined sample derived from the hydrotalcite prepared at pH 10 presented a slightly higher surface basicity and, in particular, a prominence of the basic site in the 100–150 kJ·mol^−1^ range, and associated with a higher CO_2_ adsorption capacity, when compared to the sample prepared at pH = 11. This result can be explained by the fact that at pH = 11, a small portion of the aluminum precursor could form Al(OH)_4_^−^ and not be integrated in the condensation reaction, this resulting in the enrichment of the sample in Mg, known for its basicity. This hypothesis could not be proved by the detection of Al_2_O_3_ in the calcined sample (HTCL-5 CAL), but probably the very low quantity of alumina and its presence in an amorphous or highly dispersed form can prevent its detection by XRD analysis.

This work demonstrates that the coprecipitation method in mild conditions leads to the formation of hydrotalcites with similar (sometimes higher) performances than conventional LDHs prepared by time- and energy-consuming methods. The linear correlations between the surface basicity (concentration of low- and high-energy sites) of these lamellar materials with the adsorption capacity shows their possibility to be reused several times in the same application. The possibility to tune the Mg/Al molar ratio is a promising feature to obtain a wide range of LDHs (with tuned surface basicity) that might reveal to be more efficient towards the adsorption of CO_2_ in the future.

## Figures and Tables

**Table 1:** Coprecipitation synthesis parameters of the MgAl LDHs precursors synthesized with magnesium chloride and two different aluminum sources, and different amounts of OH^−^.

Sample	Al Source	Mg/Al Ratio	NaOH Amount (mL)
HTCL-1	AlCl_3_.6H_2_O	3.02	28
HTCL-3	Al(C_5_H_7_O_2_)_3_	2.99	22
HTCL-5	AlCl_3_.6H_2_O	3.00	16

**Table 2:** TGA analyses and the weight losses of hydrotalcite precursors compared to similar materials reported in the literature.

Sample	Weight Loss 25–200 °C (%)	Weight Loss 200–600 °C (%)	Total Weight Loss (%)	Comments	Ref.
HTCL-1	17.02	26.04	43.06	-	This article
HTCL-3	13.03	31.32	44.36	-	This article
HTCL-5	16.44	25.46	41.90	-	This article
LDH1	12.7	29.3	42.00	Commercial MgAl LDH	[50]
LDH2	15.2	26.7	41.90	Commercial MgAl LDH	[50]
LDH_amm	15	38.83	53.83	Coprecipitation with ammonia	[51]
LDH_70	15	29	44.00	Coprecipitation at low supersaturation, then pretreatment at 70 °C	[52]
LDH_140	10	30	40.00	Coprecipitation at low supersaturation, then pretreatment at 140 °C	[52]
Mg/Al_LDH	24	23	47.00	Coprecipitation at low supersaturation	[53]

**Table 3:** Comparisons of the BET surface areas and CO_2_ adsorption capacities between the calcined hydrotalcite samples and similar materials reported in the literature.

Sample	Mg/Al Molar Ratio	T_pretreatment_ (°C)	T_adsorption_ (°C)	SSA (m^2^·g^−1^)	CO_2_ Adsorption Capacity (mmol·g^−1^)	Ref.
HTLC-1 CAL	3	300	200	138	0.386	This work
HTLC-3 CAL	3	300	200	180	0.527	This work
HTLC-5 CAL	3	300	200	139	0.417	This work
HTLC_Low	2	500	450	63	0.270	[61]
HTLC_High	2	500	450	154	0.260	[61]
LDO200	3	400	200	167	0.486	[62]
Htlc-200	3	200	200	66	0.604	[63]
Htlc-400	3	400	200	184	0.896	[63]
Htlc	3	-	300	74	0.5	[64]

## Data Availability

The data presented in this study are available on request from the corresponding author.

## References

[1] Wang Y., Du T., Liu L., Che S., Song Y., Fang X. A Review of Layered Double Hydroxides as Intermediate-Temperature CO_2_ Adsorbents. Proceedings of the 2017 6th International Conference on Energy, Environment and Sustainable Development (ICEESD 2017).

[2] Marquevich M., Medina F., Montané D. (2001). Hydrogen Production via Steam Reforming of Sunflower Oil over Ni/Al Catalysts from Hydrotalcite Materials. Catal. Commun..

[3] Ashok J., Subrahmanyam M., Venugopal A. (2008). Hydrotalcite Structure Derived Ni–Cu–Al Catalysts for the Production of H_2_ by CH_4_ Decomposition. Int. J. Hydrogen Energy.

[4] Li D., Wang L., Koike M., Nakagawa Y., Tomishige K. (2011). Steam Reforming of Tar from Pyrolysis of Biomass over Ni/Mg/Al Catalysts Prepared from Hydrotalcite-like Precursors. Appl. Catal. B Environ..

[5] Yong Z., Mata V., Rodriguez A. (2002). Adsorption of Carbon Dioxide at High Temperature—A Review. Sep. Purif. Technol..

[6] León M., Díaz E., Bennici S., Vega A., Ordóñez S., Auroux A. (2010). Adsorption of CO_2_ on Hydrotalcite-Derived Mixed Oxides: Sorption Mechanisms and Consequences for Adsorption Irreversibility. Ind. Eng. Chem. Res..

[7] Yang Z., Wei J., Zeng G., Zhang H., Tan X., Ma C., Li X., Li Z., Zhang C. (2019). A Review on Strategies to LDH-Based Materials to Improve Adsorption Capacity and Photoreduction Efficiency for CO_2_. Coord. Chem. Rev..

[8] Molinard A., Vansant E.F. (1995). Controlled Gas Adsorption Properties of Various Pillared Clays. Adsorption.

[9] Venaruzzo J., Volzone C., Rueda M., Ortiga J. (2002). Modified Bentonitic Clay Minerals as Adsorbents of CO, CO_2_ and SO_2_ Gases. Microporous Mesoporous Mater..

[10] Moura K.O., Pastore H.O. (2013). Comparative Adsorption of CO_2_ by Mono-, Di-, and Triamino-Organofunctionalized Magnesium Phyllosilicates. Environ. Sci. Technol..

[11] Sarker A.I., Aroonwilas A., Veawab A. (2017). Equilibrium and Kinetic Behaviour of CO_2_ Adsorption onto Zeolites, Carbon Molecular Sieve and Activated Carbons. Energy Procedia.

[12] Coudert F.-X., Kohen D. (2017). Molecular Insight into CO_2_ “Trapdoor” Adsorption in Zeolite Na-RHO. Chem. Mater..

[13] Mao G. (1993). Synthesis and CO_2_ Adsorption Features of a Hydrotalcite-Like Compound of the Mg^2+^-Al^3+^-Fe(CN)_6_^4-^ System with High Layer-Charge Density. Clays Clay Miner..

[14] Tsuji M., Mao G., Yoshida T., Tamaura Y. (1993). Hydrotalcites with an Extended Al^3+^-Substitution: Synthesis, Simultaneous TG-DTA-MS Study, and Their CO_2_ Adsorption Behaviors. J. Mater. Res..

[15] Dantas T.C.M., Junior V.J.F., dos Santos A.P.B., Bezerra F.A., Araujo A.S., Alves A.P.M. (2015). CO_2_ Adsorption on Modified Mg–Al-Layered Double Hydroxides. Adsorpt. Sci. Technol..

[16] Jiang Y., Ling J., Xiao P., He Y., Zhao Q., Chu Z., Liu Y., Li Z., Webley P.A. (2018). Simultaneous Biogas Purification and CO_2_ Capture by Vacuum Swing Adsorption Using Zeolite NaUSY. Chem. Eng. J..

[17] Yu Y., Li X., Krishna R., Liu Y., Cui Y., Du J., Liang Z., Song X., Yu J. (2018). Enhancing CO_2_ Adsorption and Separation Properties of Aluminophosphate Zeolites by Isomorphous Heteroatom Substitutions. ACS Appl. Mater. Interfaces.

[18] Bhatta L.K.G., Subramanyam S., Chengala M.D., Bhatta U.M., Venkatesh K. (2015). Enhancement in CO_2_ Adsorption on Hydrotalcite-Based Material by Novel Carbon Support Combined with K_2_CO_3_ Impregnation. Ind. Eng. Chem. Res..

[19] Moreira R.F.P.M., Soares J.L., Casarin G.L., Rodrigues A.E. (2006). Adsorption of CO_2_ on Hydrotalcite-like Compounds in a Fixed Bed. Sep. Sci. Technol..

[20] Gao Y., Zhang Z., Wu J., Yi X., Zheng A., Umar A., O’Hare D., Wang Q. (2013). Comprehensive Investigation of CO_2_ Adsorption on Mg–Al–CO_3_ LDH-Derived Mixed Metal Oxides. J. Mater. Chem. A.

[21] Radha S., Navrotsky A. (2014). Energetics of CO_2_ Adsorption on Mg–Al Layered Double Hydroxides and Related Mixed Metal Oxides. J. Phys. Chem. C.

[22] Colonna S., Bastianini M., Sisani M., Fina A. (2018). CO_2_ Adsorption and Desorption Properties of Calcined Layered Double Hydroxides. J. Therm. Anal. Calorim..

[23] Hutson N.D., Attwood B.C. (2008). High Temperature Adsorption of CO_2_ on Various Hydrotalcite-like Compounds. Adsorption.

[24] Tang N., He T., Liu J., Li L., Shi H., Cen W., Ye Z. (2018). New Insights into CO_2_ Adsorption on Layered Double Hydroxide (LDH)-Based Nanomaterials. Nanoscale Res. Lett..

[25] Choi S., Drese J.H., Jones C.W. (2009). Adsorbent Materials for Carbon Dioxide Capture from Large Anthropogenic Point Sources. ChemSusChem.

[26] Samanta A., Zhao A., Shimizu G.K.H., Sarkar P., Gupta R. (2012). Post-Combustion CO_2_ Capture Using Solid Sorbents: A Review. Ind. Eng. Chem. Res..

[27] Yu C.-H., Huang C.-H., Tan C.-S. (2012). A Review of CO_2_ Capture by Absorption and Adsorption. Aerosol Air Qual. Res..

[28] Megías-Sayago C., Bingre R., Huang L., Lutzweiler G., Wang Q., Louis B. (2019). CO_2_ Adsorption Capacities in Zeolites and Layered Double Hydroxide Materials. Front. Chem..

[29] Ram Reddy M.K., Xu Z.P., Diniz da Costa J.C. (2008). Influence of Water on High-Temperature CO_2_ Capture Using Layered Double Hydroxide Derivatives. Ind. Eng. Chem. Res..

[30] Bellotto M., Rebours B., Clause O., Lynch J., Bazin D., Elkaïm E. (1996). Hydrotalcite Decomposition Mechanism: A Clue to the Structure and Reactivity of Spinel-like Mixed Oxides. J. Phys. Chem..

[31] Tichit D., Bennani M.N., Figueras F., Ruiz J.R. (1998). Decomposition Processes and Characterization of the Surface Basicity of Cl^−^ and CO_3_^2-^ Hydrotalcites. Langmuir.

[32] Kagunya W., Hassan Z., Jones W. (1996). Catalytic Properties of Layered Double Hydroxides and Their Calcined Derivatives. Inorg. Chem..

[33] Yan K., Liu Y., Lu Y., Chai J., Sun L. (2017). Catalytic Application of Layered Double Hydroxide-Derived Catalysts for the Conversion of Biomass-Derived Molecules. Catal. Sci. Technol..

[34] Chisem I.C., Jones W., Martín I., Martín C., Rives V. (1998). Probing the Surface Acidity of Lithium Aluminium and Magnesium Aluminium Layered Double Hydroxides. J. Mater. Chem..

[35] Prinetto F., Ghiotti G., Durand R., Tichit D. (2000). Investigation of Acid−Base Properties of Catalysts Obtained from Layered Double Hydroxides. J. Phys. Chem. B.

[36] Kuśtrowski P., Chmielarz L., Bożek E., Sawalha M., Roessner F. (2004). Acidity and Basicity of Hydrotalcite Derived Mixed Mg–Al Oxides Studied by Test Reaction of MBOH Conversion and Temperature Programmed Desorption of NH_3_ and CO_2_. Mater. Res. Bull..

[37] Lei X., Zhang F., Yang L., Guo X., Tian Y., Fu S., Li F., Evans D.G., Duan X. (2007). Highly Crystalline Activated Layered Double Hydroxides as Solid Acid-Base Catalysts. AIChE J..

[38] Pérez-Barrado E., Pujol M.C., Aguiló M., Llorca J., Cesteros Y., Díaz F., Pallarès J., Marsal L.F., Salagre P. (2015). Influence of Acid–Base Properties of Calcined MgAl and CaAl Layered Double Hydroxides on the Catalytic Glycerol Etherification to Short-Chain Polyglycerols. Chem. Eng. J..

[39] Parida K., Das J. (2000). Mg/Al Hydrotalcites: Preparation, Characterisation and Ketonisation of Acetic Acid. J. Mol. Catal. A Chem..

[40] Pavel O.D., Tichit D., Marcu I.-C. (2012). Acido-Basic and Catalytic Properties of Transition-Metal Containing Mg–Al Hydrotalcites and Their Corresponding Mixed Oxides. Appl. Clay Sci..

[41] Wu Y.-J., Li P., Yu J.-G., Cunha A.F., Rodrigues A.E. (2013). K-Promoted Hydrotalcites for CO_2_ Capture in Sorption Enhanced Reactions. Chem. Eng. Technol..

[42] Hanif A., Dasgupta S., Divekar S., Arya A., Garg M.O., Nanoti A. (2014). A Study on High Temperature CO_2_ Capture by Improved Hydrotalcite Sorbents. Chem. Eng. J..

[43] Coenen K., Gallucci F., Mezari B., Hensen E., van Sint Annaland M. (2018). An In-Situ IR Study on the Adsorption of CO_2_ and H_2_O on Hydrotalcites. J. CO2 Util..

[44] Lopez T., Bosch P., Ramos E., Gomez R., Novaro O., Acosta D., Figueras F. (1996). Synthesis and Characterization of Sol−Gel Hydrotalcites. Structure and Texture. Langmuir.

[45] Bolognini M., Cavani F., Scagliarini D., Flego C., Perego C., Saba M. (2003). Mg/Al Mixed Oxides Prepared by Coprecipitation and Sol–Gel Routes: A Comparison of Their Physico-Chemical Features and Performances in m-Cresol Methylation. Microporous Mesoporous Mater..

[46] Othman M.R., Rasid N.M., Fernando W.J.N. (2006). Effects of Thermal Treatment on the Micro-Structures of Co-Precipitated and Sol–Gel Synthesized (Mg–Al) Hydrotalcites. Microporous Mesoporous Mater..

[47] Prince J., Montoya A., Ferrat G., Valente J.S. (2009). Proposed General Sol−Gel Method to Prepare Multimetallic Layered Double Hydroxides: Synthesis, Characterization, and Envisaged Application. Chem. Mater..

[48] Smalenskaite A., Vieira D.E.L., Salak A.N., Ferreira M.G.S., Katelnikovas A., Kareiva A. (2017). A Comparative Study of Co-Precipitation and Sol-Gel Synthetic Approaches to Fabricate Cerium-Substituted Mg Al Layered Double Hydroxides with Luminescence Properties. Appl. Clay Sci..

[49] Cavani F., Trifiro F., Vaccari A. (1991). Hydrotalcite-Type Anionic Clays: Preparation, Properties and Applications. Catal. Today.

[50] Ardanuy M., Velasco J.I. (2011). Mg–Al Layered Double Hydroxide Nanoparticles. Appl. Clay Sci..

[51] Mondal S., Dasgupta S., Maji K. (2016). MgAl- Layered Double Hydroxide Nanoparticles for Controlled Release of Salicylate. Mater. Sci. Eng. C.

[52] Sharma S.K., Kushwaha P.K., Srivastava V.K., Bhatt S.D., Jasra R. (2007). V Effect of Hydrothermal Conditions on Structural and Textural Properties of Synthetic Hydrotalcites of Varying Mg/Al Ratio. Ind. Eng. Chem. Res..

[53] Klemkaite K., Prosycevas I., Taraskevicius R., Khinsky A., Kareiva A. (2011). Synthesis and Characterization of Layered Double Hydroxides with Different Cations (Mg, Co, Ni, Al), Decomposition and Reformation of Mixed Metal Oxides to Layered Structures. Open Chem..

[54] Thommes M., Kaneko K., Neimark A.V., Olivier J.P., Rodriguez-Reinoso F., Rouquerol J., Sing K.S.W. (2015). Physisorption of Gases, with Special Reference to the Evaluation of Surface Area and Pore Size Distribution (IUPAC Technical Report). Pure Appl. Chem..

[55] Balsamo N., Mendieta S., Oliva M., Eimer G., Crivello M. (2012). Synthesis and Characterization of Metal Mixed Oxides from Layered Double Hydroxides. Procedia Mater. Sci..

[56] Reyero I., Velasco I., Sanz O., Montes M., Arzamendi G., Gandía L.M. (2013). Structured Catalysts Based on Mg–Al Hydrotalcite for the Synthesis of Biodiesel. Catal. Today.

[57] Kurnia Julianti N., Kusuma Wardani T., Gunardi I., Roesyadi A. (2017). Effect of Calcination at Synthesis of Mg-Al Hydrotalcite Using Co-Precipitation Method. J. Pure Appl. Chem. Res..

[58] Auroux A. (1997). Acidity Characterization by Microcalorimetry and Relationship with Reactivity. Top. Catal..

[59] Bennici S., Auroux A., Hargreaves S.J., Jackson S.D. (2009). Thermal analysis and calorimetric methods. Metal Oxide Catalysis.

[60] Occelli M.L., Olivier J.P., Auroux A., Kalwei M., Eckert H. (2003). Basicity and Porosity of a Calcined Hydrotalcite-Type Material from Nitrogen Porosimetry and Adsorption Microcalorimetry Methods. Chem. Mater..

[61] Yang J.-I., Kim J.-N. (2006). Hydrotalcites for Adsorption of CO_2_ at High Temperature. Korean J. Chem. Eng..

[62] Ram Reddy M.K., Xu Z.P., (Max) Lu G.Q., Diniz da Costa J.C. (2006). Layered Double Hydroxides for CO_2_ Capture: Structure Evolution and Regeneration. Ind. Eng. Chem. Res..

[63] Hutson N.D., Speakman S.A., Payzant E.A. (2004). Structural Effects on the High Temperature Adsorption of CO_2_ on a Synthetic Hydrotalcite. Chem. Mater..

[64] Yong Z., Mata, Rodrigues A.E. (2001). Adsorption of Carbon Dioxide onto Hydrotalcite-like Compounds (HTlcs) at High Temperatures. Ind. Eng. Chem. Res..

